# Formation and Evolution Mechanism of Intermetallic Compounds of Friction Stir Lap Welded Steel/Aluminum Joints

**DOI:** 10.3390/ma16186118

**Published:** 2023-09-07

**Authors:** Yongzhi Liu, Qiu Pang, Zhichao Zhang, Lan Hu

**Affiliations:** 1 Hubei Longzhong Laboratory, Wuhan University of Technology, Xiangyang 441000, China; 2 Hubei Key Laboratory of Advanced Technology for Automotive Components, Wuhan University of Technology, Wuhan 430070, China; 3 Hubei Engineering Research Center for Green & Precision Material Forming, Wuhan University of Technology, Wuhan 430070, China; 4 Department of Mechanical and Electrical Engineering, Wuhan Donghu University, Wuhan 430212, China; 5 Shanghai Aerospace Equipments Manufacturer Co., Ltd., Shanghai 200245, China

**Keywords:** friction stir lap welding, intermetallic compounds, microstructure evolution, simulation

## Abstract

Interfacial layers with brittle intermetallic compounds (IMC) greatly influence the performance of steel–aluminum friction stir lap welding (FSLW) joints. Thus, the formation and evolution of IMC between 7075-T6 aluminum alloy and galvanized DP590 steel in steel–aluminum FSLW joints were investigated. An FSLW numerical model was developed using the computational fluid dynamics method to analyze the interface temperature between the aluminum alloy and steel. Scanning electron microscopy (SEM) was conducted to observe the microstructure characterization and measure the IMC thickness. Phases among different joint zones were analyzed by X-ray diffraction (XRD) and energy dispersive spectroscopy (EDS). IMC layer formation was predicted by the effective Gibbs free energy model presented in this paper according to thermodynamic principles. The Monte Carlo method was utilized to predict the thickness of IMC layers. It was found that the IMC layer at the interface of the welded joint is composed of Fe_2_Al_5_, FeAl_3_, and Al-Zn eutectic. The IMC thickness decreased from 4.3 μm to 0.8 μm with the increasing welding speed, which was consistent with the Monte Carlo simulation results.

## 1. Introduction

The automobile lightweight is one of the hot spots in the automobile manufacturing industry. The steel–aluminum integrated body frame structure realizes the lightweight and high strength of the body frame structure by using aluminum alloy material to replace steel components in the traditional body frame steel structure [1]. The steel–aluminum composite structure can not only consider the lightweight and cost requirements but also give play to the strength advantages of high-strength steel and the weight reduction and energy absorption advantages of aluminum alloy [2]. Although there are many methods used to connect aluminum alloy to steel, including MIG arc brazing [3], laser welding [4], self-stamping riveting [5], and other technologies, to further improve the reliability of aluminum steel joints, the solid-state welding method of friction stir welding (FSW) is preferred [6].

During the welding process, solid phase diffusion of Fe and Al atoms occurs between base materials under the welding thermal cycle and severe plastic deformation. An intermetallic compound (IMC) is formed at the joint overlap interface, achieving metallurgical bonding between steel and aluminum alloy. It is clear that IMC has a significant role in achieving steel/aluminum metallurgical bonding and affecting joint performance [7,8,9]. The intervention of the Zn coat promoted the formation of an Al–Zn eutectic structure with a low melting point at the interface, which significantly improved the weldability of aluminum alloy and steel [10]. Furthermore, Zn coatings on steel were found to be beneficial for the formation of intermetallic reaction zones in solid/liquid joining procedures [11].

Both IMC composition and thickness have a significant impact on metallurgical bonding strength, which in turn determines joint performance [6]. According to the content of the element, IMC can be divided into Fe-rich IMC (FeAl, Fe_3_Al) and Al-rich IMC (FeAl_2_, Fe_2_Al_5_, and FeAl_3_). There are significant differences in performance between the two different types of IMCs, with Fe-rich IMC exhibiting better toughness, while Al-rich IMC is a brittle hard phase. Therefore, Fe-rich IMCs are more conducive to improving the metallurgical bonding strength of joints [8]. Due to significant differences in thermal cycling and plastic deformation experienced by different regions of the overlap interface during the welding process, there are usually two or more different components of IMC at the interface [6,8], and the IMC composition is influenced by welding temperature and element concentration. At present, some studies [12,13] find that the hard and brittle nature of IMC has an adverse impact on joint performance, and the thickness of IMC in steel/aluminum joints should be minimized as much as possible. However, some studies [14,15,16] find that there is a critical value for the thickness of IMC in steel/aluminum joints. When the thickness of IMC is thick, due to its hard and brittle properties, cracks are prone to occur under residual welding stress, which has a negative impact on the performance of the joint. However, when the thickness of IMC is below the critical value, the presence of IMC will not harm the strength of the joint and can even improve the performance of the joint. However, there are relatively few studies on the formation and evolution mechanism of IMC at the interface of steel/aluminum FSLW joints.

This article focuses on the unclear formation and evolution mechanism of IMC in the FSLW process of the 7075-T6 aluminum alloy and galvanized DP590 steel. The FSLW numerical simulation model based on computational fluid dynamics was established to clarify the temperature distribution of the joint. The microstructure of the joint interface was analyzed through OM and SEM. XRD and EDS were utilized to figure out the composition of IMC. Based on the temperature distribution of the joint during the welding process and the changes in Gibbs free energy, the formation order and evolution of IMCs were studied to clarify the formation mechanism of intermetallic compounds, while the IMC thickness was simulated by the Monte Carlo method.

## 2. Materials and Methods

### 2.1. Materials and Experiment

**Figure 1:**
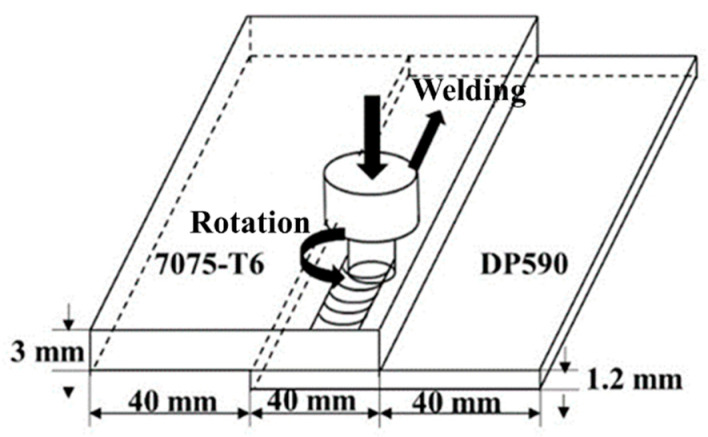
Schematic representation of the FSLW for dissimilar joining between the Al alloy and steel.

**Figure 2:**
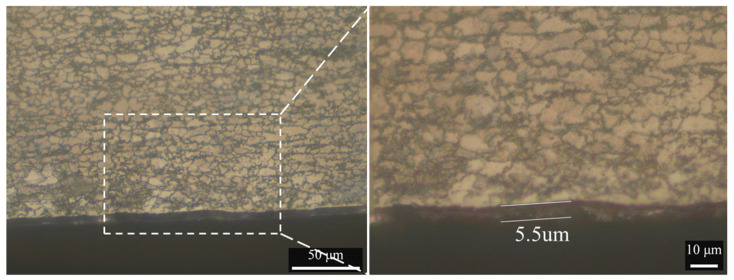
The cross-section view of DP590 galvanized steel.

The base materials used in the present study were a 3 mm thick 7075-T6 aluminum alloy sheet and a 1.2 mm thick galvanized DP590 steel sheet. Figure 1 shows a schematic illustration of the plate arrangement in the lap configuration before welding. A welding tool (made of H13 die steel) with a smooth pin was adopted in the experiment. The shoulder diameter of the tool is 12 mm, and the conical pin has a length of 2.9 mm. The root and tip diameter of the pin is 5 mm and 3.6 mm. The tilt angle of the tool was set at 2.5 degrees. As shown in Figure 2, the zinc coating in steel sheet is uniform and continuous with average thickness of 5.5 μm.

After welding, the joint was cross-sectioned perpendicular to the welding direction. The microstructure of the cross-sectioned joint was analyzed using optical microscopy (Zeiss Scope A1, Zeiss, Oberkochen, Germany) and scanning electron microscopy (SEM, JEM-7500F, JEOL, Tokyo, Japan). Energy dispersive spectrometer (EDS) analysis was carried out to determine the element distribution at the joint interfaces. The phase composition of the specimen was analyzed by X-ray diffraction (XRD, Empyrean). The cross-sections of the metallographic specimens were polished and etched with Keller’s reagent (2.5 mL HF, 1 mL HCl, 1.5 mL HNO_3_, and 95 mL H_2_O) and 4% nitric acid alcohol for the aluminum alloy and steel, respectively.

### 2.2. Simulations

#### 2.2.1. Friction Stir Lap Welding Simulation Model

**Figure 3:**
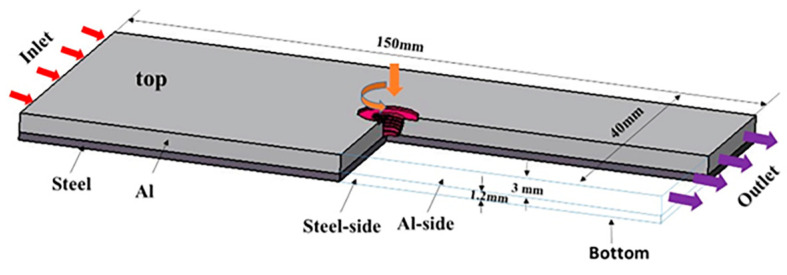
The computational domain and its dimensions.

An FSLW numerical model was developed using the computational fluid dynamics method in ANSYS/FLUENT to analyze the temperature distribution under the steady-state welding of the 7075-T6 aluminum alloy and DP590 steel under the conditions listed in Table 1. Only the overlap area of workpieces was set as computational domain, which is illustrated in Figure 3. The swirling process of stirring pin was not taken into consideration in the model.

To model the dissimilar FSLW of 7075 aluminum alloy and DP590 steel, the material is treated as an incompressible non-Newtonian fluid, with a viscoplastic flow [7]. The material flow constitutive equation is expressed by the hyperbolic sinusoidal relation proposed by Sellar and Tegart [17].

(1) $$\bar{\dot{\varepsilon }}=A{\left(\sinh\alpha \sigma \right)}^{n}\exp(-\frac{Q}{RT}),$$

where $\bar{\dot{\varepsilon }}$ is the effective strain rate, σ is the flow stress, *Q* is the thermal activation energy of deformation, *R* is the gas constant, *T* is the temperature, n is the stress index, A and α are constants related to the material. The material constitutive parameters are listed in Table 2 [18,19,20].

During the FSLW process, the heat generation of the contact surface between the workpiece and the tool involves friction heat generation and plastic deformation heat generation [18]. In this model, both heat inputs from friction and plastic deformation were taken into consideration. The heat generation rates at the contact surface between the shoulder/pin and the workpiece are defined by Equations (2)–(4) [7,21], respectively.

(2) $$q_{sb}\left(r\right)=\left[\eta \left(1-\delta \right)\tau_{c}+\delta \mu_{f}P_{N}\right]\left(\omega r-u_{weld}\sin\theta \right),$$

(3) $$q_{pb}\left(r\right)=\left[\eta \left(1-\delta \right)\tau_{c}+\delta \mu_{f}P_{N}\right]\left(\omega r-u_{weld}\sin\theta \right),$$

(4) $$q_{ps}\left(r\right)=\left[\eta \left(1-\delta \right)\tau_{c}+\delta \mu_{f}\sigma_{y}\right]\left(\omega r-u_{weld}\sin\theta \right),$$

where $q_{sb}\left(r\right)$, $q_{pb}\left(r\right)$, and $q_{ps}\left(r\right)$ represent the heat generation rates of the shoulder bottom, the pin bottom, and the pin side surface; *η* is the conversion efficiency of plastic deformation; δ is the slip coefficient of the interface; $\tau_{c}$ is the shear yield stress of plastic material;$\;\mu_{f}$ is the friction coefficient of the stirring pin and the workpiece contact surface; $\;\;P_{N}$ is the axial pressure of the stirring pin, which is equal to 45 MPa; $\sigma_{y}$ is the yield strength of the workpiece material; ω  is the welding speed; *r* represents the distance from the elemental area to the tool axis; $u_{weld}$ is the material flow speed; θ is the angle between the welding direction and the *r* radial vector direction. $\delta_{Al}\;$ = 0.4, $\delta_{Steel}$ = 0.7, $\mu_{f_{Al}}\;$ = 0.35, $\mu_{f_{steel}}$ = 0.3.

In addition, the plastic deformation heat source in the shear zone was also taken into consideration, which can be expressed by [7,21]

(5) $$S_{v}=f_{m}\mu \Phi ,$$

where $f_{m}$ is an arbitrary constant that represents the extent of atomic mixing in the system, and Φ is given by [7,18]

(6) $$\Phi =2\left[{\left(\frac{\partial u}{\partial x}\right)}^{2}+{\left(\frac{\partial v}{\partial y}\right)}^{2}+{\left(\frac{\partial w}{\partial z}\right)}^{2}\right]+{\left(\frac{\partial u}{\partial y}+\frac{\partial v}{\partial x}\right)}^{2}+{\left(\frac{\partial u}{\partial z}+\frac{\partial w}{\partial x}\right)}^{2}+{\left(\frac{\partial w}{\partial y}+\frac{\partial v}{\partial z}\right)}^{2},$$

where *u*, *v*, and *w* are the velocity components of material flow in the *x*-, *y*-, and *z*-directions, respectively. The relative velocity at the contact area between the shoulder/pin and the workpiece can be expressed by Equations (7)–(9) [22].

(7) $$u=\left(1-\delta \right)\left(\omega r\sin\theta -u_{weld}\right),$$

(8) v=(1−δ)ωrcosθ,

(9) $$w=\frac{\psi \omega }{2\pi },$$

where ψ is the thread pitch.

The convection heat transfer coefficient on the side and bottom surfaces of the workpiece that contacts the backing plate and clamping equipment was defined as 500.0 W/(m^2^·K) [7], the top surface of the workpiece was taken as a free surface contacting with air, and the convection heat transfer coefficient on the top surface was defined as 30.0 W/(m^2^·K) [7].

#### 2.2.2. Monte Carlo Simulation Model

The Monte Carlo simulation model was developed in MATLAB code. The microstructure is generated based on a two-dimensional N × N array of a uniform-sized square cell (lattice site). *N* is the number of the discretized lattice points in two directions. To describe each lattice site, parameter *q*, which represents the crystallographic orientation of the lattice site (ranging from 1 to *Q*), is used. The initial lattice properties are set so that the lattice site belongs to Al (*q* = 0) and otherwise belongs to Fe (*q* = 1).

In this paper, the chemical-free energy item and the boundary energy term are introduced into Hamiltonian of the system in the *q*-state Potts model as follows:

(10) $$H=\frac{1}{2}\sum_{i}^{n}\sum_{j}^{m}G_{S_{i}S_{j}}^{b}+\sum_{i}^{n}G_{i}^{c},$$

where $G_{i}^{c}$ is the chemical-free energy of cell *i* with its orientation *S_i_*; $G_{S_{i}S_{j}}^{b}$ is the boundary energy between MC cell *i* and cell *j* with their corresponding orientations.

The boundary energy of the grain boundary between two neighboring cells (cell *i* and cell *j*) can be expressed with the Read–Shockley model as follows [23]:

(11) $$\gamma =\gamma_{m}\left(\frac{\theta }{\theta_{m}}\right)\left(1-\ln\frac{\theta }{\theta_{m}}\right),$$

where *γ* is the interface energy, *θ* is the orientation angle of grain boundary dislocation; $\gamma_{m}=0.56J\cdot m^{-2}$, $\theta_{m}=15^{\circ }$ [24]. When the grain boundary becomes a high-angle grain boundary, the energy of the grain boundary has nothing to do with the direction and angle of the dislocation.

In each Monte Carlo step (MCS), cell *i* is selected at random, and then neighbors of the selected lattice site and their crystallographic orientations were determined. One neighbor site was chosen at random out of the eight neighbors. If there is a certain proportion of Fe and Al in the nine lattices centered on *i*, there is a certain probability that a phase will form. If the selected lattice site (*i*) and chosen neighboring site (*j*) belong to two different phases, the chosen neighboring site orientation (*q_j_*) will be exchanged with the first selected lattice site orientation (*q_i_*). Calculate the energies of the selected lattice and chosen neighboring sites (*H*_1_) at the initial state, using Equation (10). Calculate the energies of the selected lattice and chosen neighboring sites (*H*_2_) after reorientation or orientation exchanges, using Equation (10). Calculate the net energy change (Δ*H* = *H*_2_ − *H*_1_) before and after reorientation or orientation exchanges. The new orientation assignment or exchange is accepted with a transition probability, *P_x_*, of [25,26]:

(12) $$P_{x}=\{\begin{array}{cc} 1 & \Delta H<0\\ \exp\left(-\frac{\Delta H}{k_{B}T}\right) & \Delta H>0 \end{array},$$

where *T* is the simulation temperature, *k_B_* is the Boltzmann’s constant. Generate a random probability *R* between 0 and 1. The reorientation is accepted if *R* ≤ *P_x_*, otherwise the lattice site orientation is unchanged.

## 3. Results and Discussion

### 3.1. Temperature Distribution and Microstructure Morphology

**Figure 4:**
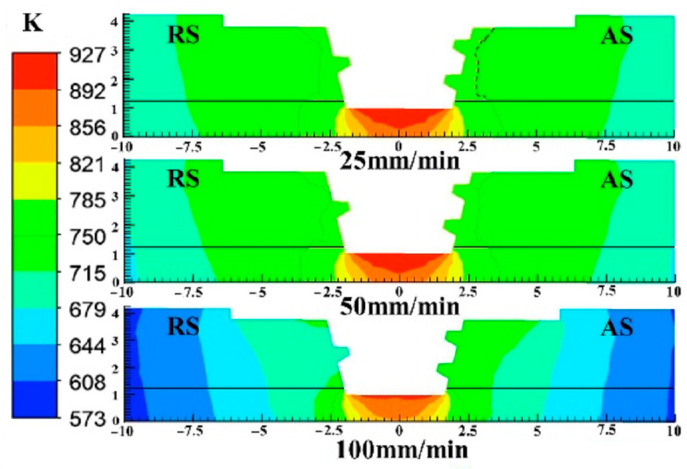
Temperature distribution at transverse cross−section with different welding speeds.

Figure 4 shows the simulation results of temperature distribution at transverse cross-sections with different welding speeds. The maximum temperature point appears at the contact surface between the stirring pin and the steel. The flow stress of steel is greater than aluminum alloy, which accounts for higher heat production from plastic deformation in the welding process.

**Figure 5:**
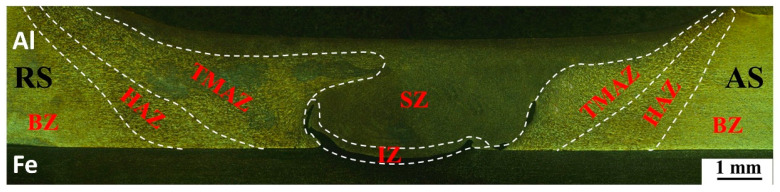
Morphology of steel–aluminum FSLW joint.

Figure 5 shows the macroscopic morphology of a typical steel–aluminum FSLW joint under the parameter of 1200 r/min–100 mm/min. The joint cross-section can be divided into the stirring zone (SZ), interface zone (IZ), thermo-mechanical affected zone (TMAZ), heat affected zone (HAZ), and base material zone (BZ). Under the mechanical and thermal effects, severe plastic deformation is produced in the SZ. Meanwhile, the material in the SZ reaches a good plastic flow state, and the material within a certain range near the stirring pin is stirred, resulting in a significantly wider SZ than the diameter of the stirring pin. During the welding process, the TMAZ does not come into direct contact with the welding tool, resulting in a low degree of plastic deformation. The TMAZ shows a significant difference between the advancing side and the retreating side of the joint, and the boundary between the advancing side and the SZ is more obvious. This is because, during the welding process, the material shear rate on the advancing side is relatively high. The structure of the HAZ only undergoes welding thermal cycles without any mechanical effect, and the area is also not affected by material plastic flow.

### 3.2. Microstructure Characterization

**Figure 6:**
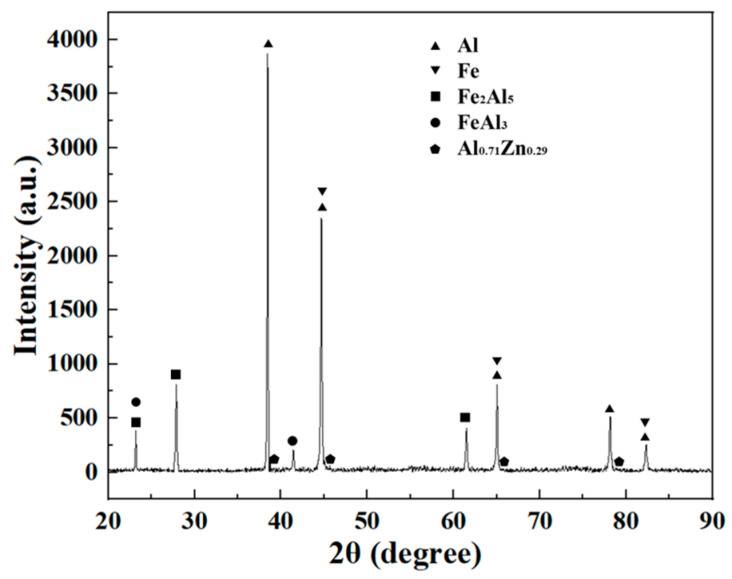
XRD results of the joint interface.

**Figure 7:**
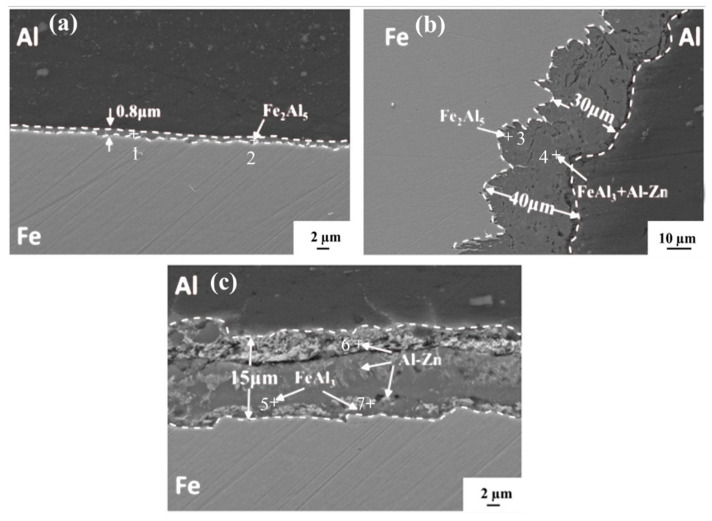
IMC distribution in different regions (**a**) IZ, (**b**) TMAZ, (**c**) HAZ.

According to the XRD results in Figure 6, the IMCs of the galvanized steel–Al joint were determined to be Fe_2_Al_5_, FeAl_3_, and the Al-Zn eutectics. Figure 7 shows the IMC in different areas of a typical joint. During the steel/aluminum FSLW process, the Al and Fe atoms in the bonding area of the lap interface undergo solid-phase diffusion, and a continuum Al-Fe IMC layer was formed due to welding heat and plastic deformation. The results of the EDS point analysis are shown in Table 3. During the welding process, the Zn coating at the interface of the IZ is extruded by the stirring pin. The interface IMC in the IZ is generated by the solid phase diffusion of Al and Fe atoms. Its composition is only Fe_2_Al_5_ with a thickness of 0.8 µm (Figure 7a). The welding pressure on the interface of the TMAZ is relatively small, and there are some remaining Al-Zn eutectic components at the interface of this area, and the presence of Zn can promote the growth of Al-Fe IMC, so the IMC thickness of the TMAZ is 30~40 μm (Figure 7b). From the results of EDS point scanning, there are three components of Fe_2_Al_5_, FeAl_3_, and Al-Zn eutectic at the same time in IMC. The IMC distribution at the interface of the HAZ of the joint is shown in Figure 7c. Compared with TMAZ, the IMC thickness is reduced to only 15 μm. The results show that the IMC in the HAZ is divided into two layers, the upper layer is Al-Zn eutectic composition, and the lower layer is mainly the mixture of FeAl_3_ and Al-Zn eutectic composition.

### 3.3. Interfacial Layer Formation and Evolution

According to the effective heat of formation (EHF) model proposed by Pretorius et al. [25], the effective Gibbs free energy of formation can be obtained by analogy. The formation of primary phases of the Al-Fe binary system on the reaction and the order of formation of each phase can be predicted.

(13) $$\Delta G^{’}\left(T\right)=\frac{c_{eff}}{c_{0}}\Delta G_{m}\left(T\right),$$

where ∆*G′*(*T*) is the change in effective Gibbs free energy of formation at different temperatures, ∆*G_m_* (*T*) is Moore Gibbs free energy change at different temperatures, *c_eff_* is the effective concentration of the limiting element on the reaction interface, *c*_0_ is the concentration of the limiting element in the compound. Since the effective concentration of elements at the reaction interface is difficult to obtain, the eutectic component at the lowest eutectic temperature in the reaction system is usually selected as the effective concentration [26]. For the Al-Fe binary system, according to the Al-Fe binary phase diagram, the effective concentration of Al Fe at the lowest eutectic temperature (928 K) is 99.1% and 0.9%, respectively.

The molar Gibbs free energy of the Al-Fe system can be expressed by [27]:

(14) $$G_{m}=G_{m}^{srf}+G_{m}^{cfg}+G_{m}^{E}=\sum_{i}x_{i}G_{i}\left(T\right)+RT\sum_{i}x_{i}\ln\left(x_{i}\right)+x_{i}x_{j}\sum_{v}L_{ij}{\left(x_{i}-x_{j}\right)}^{v}$$

where $G_{m}^{srf}$, $G_{m}^{cfg}$ and $G_{m}^{E}$ are the surface of reference, configurational, and the excess contributions, respectively; *x_i_* is the molar fraction of component i; *G_i_*(T) represents the Gibbs energy of component *i*, which is a function of the temperature; R is the gas constant; T is the absolute temperature; *L_ij_* is the interaction parameter between components i and j; and the *L_ij_* and *G_i_*(T) values of Fe and Al are listed in Table 4 [7,27].

For the reaction $\mathrm{xFe}+\mathrm{yAl}={\mathrm{Fe}}_{x}{\mathrm{Al}}_{y}$ in the Al–Fe system, the Gibbs free energy change can be defined as:

(15) $$\Delta G=G_{m_{Fe_{x}Al_{y}}}-xG_{Fe}-yG_{Al},$$

According to the Al–Fe phase diagram [27], there are five possible components in the IMC layers: FeAl, Fe_3_Al, FeAl_2_, FeAl_3_, and Fe_2_Al_5_. Therefore, the Gibbs free energy change in the Al-Fe intermetallic compounds can be described by Equations (16)–(20), according to Equations (13)–(15):

(16) $$\Delta G_{m}{}_{{}_{FeAl}}\left(T\right)=-33490.691-345.968T+55.262\mathrm{TlnT}-0.0141\times T^{2}-151451\times T^{-1}$$

(17) $$\Delta G_{m}{}_{{}_{Fe_{3}Al}}\left(T\right)=-59480.674-461.42T+76.72\mathrm{TlnT}-0.004\times T^{2}-229616.75\times T^{-1}$$

(18) $$\Delta G_{m}{}_{{}_{FeAl_{2}}}\left(T\right)=-19813.351-380.803T+58.01\mathrm{TlnT}-0.0128\times T^{2}-150362\times T^{-1}$$

(19) $$\Delta G_{m}{}_{{}_{FeAl_{3}}}\left(T\right)=-7552.39-593.22T+89.07\mathrm{TlnT}-0.0384\times T^{2}-224726.25\times T^{-1}$$

(20) $$\Delta G_{m}{}_{{}_{Fe_{2}Al_{5}}}\left(T\right)=-6242.03-1167.51T+176.37\mathrm{TlnT}-0.072\times T^{2}-450152.57\times T^{-1}$$

**Figure 8:**
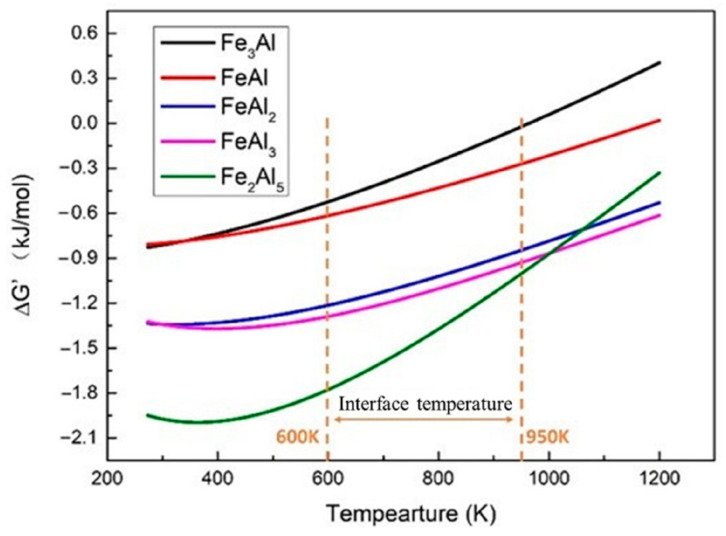
Values of ∆*G′* at different temperatures.

Figure 8 shows the ∆*G*′ of each Al-Fe IMC at the corresponding concentration of the lowest eutectic point at different temperatures. In the welding temperature range of 600 K~950 K, the ∆*G*′ of Fe_2_Al_5_ is the smallest, so Fe_2_Al_5_ is the first to be formed at the joint interface, while FeAl_3_ is formed after Fe_2_Al_5_. In the welding process, Fe_2_Al_5_ first nucleates and grows at the lap interface. When the continuous Fe_2_Al_5_ layer is formed, the diffusion of the Al and Fe atom is hindered, resulting in the decrease in the Fe atom concentration on the side of the Fe_2_Al_5_ layer away from the steel, and the growth of Fe_2_Al_5_ is inhibited. Meanwhile, the concentration of the Al atom on this side is relatively high, so FeAl_3_ is gradually formed on this side, and excessive Al can also react with Fe_2_Al_5_ to form FeAl_3_ [28].

**Figure 9:**
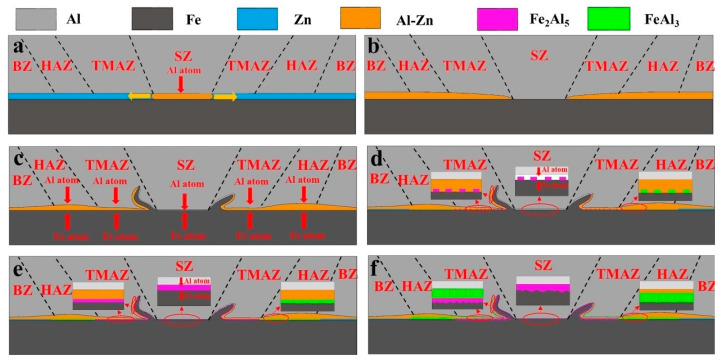
IMC formation and evolution mechanism. (**a**–**f**) describes the IMC formation and evolution order through the FSLW process.

In the steel/aluminum FSLW process, the IMC formation and evolution mechanism of the joint interface are shown in Figure 9. The formation process includes IMC nucleation and growth. First, as shown in Figure 9a, the temperature in the area in front of the stirring pin gradually rises under the effect of heat conduction, and the diffusion of various elements in this area gradually increases. When the temperature reaches 654 K, the zinc-rich coating on the surface of the steel plate reacts with Al atoms diffused to the interface to form a low melting point Al-Zn eutectic composition. The melted Al-Zn eutectic component is extruded by the stirring pin from the IZ and flows to other areas of the joint. While the welding pressure and temperature of the TMAZ and HAZ are lower than that of IZ, there remain some Al-Zn eutectic components, as shown in Figure 9b. As the welding process continues (Figure 9c), a hook-like structure will form at the interface. The aluminum alloy and the steel at the interface of the IZ are in direct contact with each other in a high-temperature and high-strain environment. When Al and Fe atoms start solid-phase diffusion, the element concentration at the joint interface gradually increases. With the distance increasing from the IZ, the plastic deformation and the temperature of the material gradually decrease, and the element diffusion effect is weakened. Therefore, the Al and Fe elements in the TMAZ and HAZ are not as concentrated as those in the IZ.

As the diffusion of atoms progresses (Figure 9d), a solid solution structure is formed at the interface of the joint. When the diffusion reaches a certain level, the solid solution at the interface transforms into a supersaturated state. In this state, a stable phase compound will be formed at the interface. In the Al-Fe system, the reaction always proceeds in the direction of the decrease in Gibbs free energy, that is, the lower the Gibbs free energy of the compound, the easier it is to form. Therefore, Fe_2_Al_5_ first nucleates at the interface between the IZ and TMAZ. At the interface of the HAZ, FeAl_3_ is the first to nucleate due to the limitation of element concentration. After the formation of the IMC crystal nucleus, the solute atoms in the solid solution at the interface precipitate out of the supersaturation state. When Al and Fe atoms continue to diffuse to the interface of the joint, the solid solution transforms into a supersaturated state again, causing the IMC nucleus to grow, and the IMC nucleus continues to be generated at the higher grain boundary energy, and finally, IMC grows along with the interface and forms a continuous IMC layer (Figure 9e).

The formation of a continuous IMC layer at the interface of the IZ blocks the mutual contact between the steel and aluminum alloy (Figure 9f), and the interface changes from the Al-Fe interface to the Al-Fe_2_Al_5_, Fe-Fe_2_Al_5_ interfaces. At this time, the growth of IMC is mainly affected by the influence of the diffusion rate of Al and Fe atoms. Since the diffusion rate of Fe atoms in the Fe_2_Al_5_ layer is much lower than that of Al atoms, the growth of Fe_2_Al_5_ mainly occurs at the Fe-Fe_2_Al_5_ interface. In the TMAZ, the presence of the Fe_2_Al_5_ layer hinders the diffusion of Fe atoms to the aluminum side, resulting in a lower concentration of Fe atoms in the Fe_2_Al_5_ layer near the aluminum side, so that the Al-rich phase FeAl_3_ is formed on this side and is mixed with the Al-Zn eutectic component. Finally, a dense Fe_2_Al_5_ layer is formed at the interface of the IZ of the joint, and the IMC of the TMAZ is divided into two layers, namely the Fe_2_Al_5_ layer and a mixed layer of FeAl_3_ + Al-Zn eutectic composition, and the IMC of the HAZ is divided into Al -Zn eutectic and FeAl_3_ + Al-Zn eutectic composition mixed layer.

### 3.4. IMC Thickness

In the FSLW process, the growth of IMC at the lap interface is closely related to the welding parameters. The formation and growth of IMC are determined by the solid phase diffusion of Fe and Al atoms. The change in welding parameters can cause changes in the welding heat input, and cooling rate, which will cause changes in the diffusion of Al and Fe atoms, and ultimately have an impact on the growth of IMC. Therefore, combining the atomic diffusion process and IMC growth kinetics, the growth of the IMC layer at the joint interface is analyzed. Although there are different regions in the welding joint, the complexity of IMC composition at the interface of HAZ and TMAZ leads to uncertainty in the growth of IMC, and the IZ plays an important role in mechanical properties such as tensile strength and shear strength. Therefore, only the growth process of the IMC layer in the bonding interface area of the IZ is simulated.

According to the mechanism of IMC formation, the formation of IMC includes nucleation and growth. In the nucleation stage, the reaction rate between Al and Fe atoms is the main factor limiting the nucleation of IMC. In the planar growth process of IMC, the diffusion of Al and Fe atoms is inhibited by the Fe_2_Al_5_ layer. The growth of IMC is limited by the diffusion rate of Al and Fe atoms.

**Figure 10:**
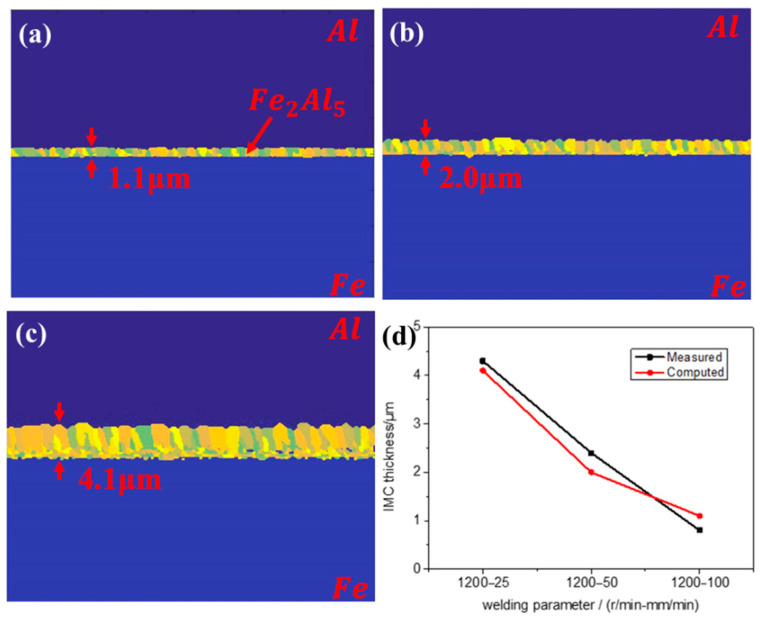
Simulation results of IMC under different welding parameters (**a**–**c**) and IMC thickness between experiment and simulation (**d**).

Figure 10 shows the interface morphology of numerical simulation and the experimental and simulated IMC thickness changes under different welding parameters. The experimental results can be viewed in our previous work [2]. With the increase in welding speed, the welding heat input decreases, the diffusion between atoms weakens, and the growth of IMC is inhibited, resulting in a decrease in IMC thickness. There is a certain difference between the simulated values and the experimental values. In this model, only the grain boundary energy and Gibbs free energy are considered, while the deformation energy is not taken into consideration. Meanwhile, the atomic diffusion rate is set as the function of the distance from the initial steel–aluminum interface fails to accurately consider the actual physical diffusion effect.

## 4. Conclusions

(1) In the process of FSLW of steel and aluminum alloy, due to the friction heat and plastic deformation, the composition of the IMC layer in the lap interface of the joint is complex, including Fe_2_Al_5_, FeAl_3_, and Al-Zn eutectic.
(2) The effective Gibbs free energy change indicates that Fe_2_Al_5_ is the first phase formed in the Al-Fe system in the welding temperature range of the joint interface between 600 K and 950 K.
(3) During the welding process, when the continuous Fe_2_Al_5_ layer is formed at the IZ, the diffusion of Al and Fe atoms is hindered, so the growth of IMC in the IZ is inhibited, and the thickness of IMC is relatively thin. However, due to the weak plastic deformation of the matrix in the TMAZ and the existence of the Fe_2_Al_5_ layer, the diffusion of the Fe element is greatly inhibited, resulting in the formation and growth of the FeAl_3_ layer near the side of the Fe_2_Al_5_ layer to the aluminum material. In addition, the presence of Zn elements promotes the growth of the IMC in the TMAZ. Due to the low welding temperature and no plastic deformation in the HAZ, resulting in weak element diffusion, the overlapping interface only forms FeAl_3_ and a hybrid layer with Al-Zn eutectic composition.
(4) The simulation results of the IMC thickness in the IZ are relatively close to the experimental results. The Monte Carlo model can predict the IMC thickness in the IZ within a certain parameter range.

## Figures and Tables

**Table 1:** The welding parameters for FSLW.

Rotational Speed (rpm)	Welding Speed (mm/min)	Depth of Penetration (mm)
1200	25/50/100	1.2

**Table 2:** Material constitutive parameters [18,19,20].

Materials	*α*	*A*	*n*	*Q*
7075-T6	1.03 × 10^9^	0.0141	5.41	129,000
DP590	3.612 × 10^13^	0.00755	6.897	329,000

**Table 3:** EDS results of each point in Figure 7 (at%).

Points	Al	Fe	Zn	Value
1	71.50	28.17	0.33	Fe_2_Al_5_
2	72.95	26.11	0.94	Fe_2_Al_5_
3	70.36	28.53	1.11	Fe_2_Al_5_
4	68.58	23.06	8.36	FeAl_3_ + Al-Zn
5	75.29	24.55	0.16	FeAl_3_
6	85.32	0.91	13.77	Al-Zn
7	67.45	24.86	7.69	FeAl_3_ + Al-Zn

**Table 4:** Computational parameters in the Gibbs free energy change [7,27].

Symbol	Formula/Value
$G_{Al}$	$-12061.144+219.68T-41.75T\ln T+0.01853\times T^{2}+74092T^{-1}$
$G_{Fe}$	$22557.71+131.817T-23.5143T\ln T-0.0043975\times T^{2}+77359T^{-1}$
$L_{Fe,Al}^{0}$	−91976.5+22.1314T
$L_{Fe,Al}^{1}$	−5672.58+4.8728T
$L_{Fe,Al}^{2}$	121.9

## Data Availability

Data sharing not applicable.
